# A comparison of foot arch measurement reliability using both digital photography and calliper methods

**DOI:** 10.1186/1757-1146-3-14

**Published:** 2010-07-14

**Authors:** Michael B Pohl, Lindsay Farr

**Affiliations:** 1 Faculty of Kinesiology, University of Calgary, AB, Canada; 2 Running Injury Clinic, University of Calgary, AB, Canada

## Abstract

**Background:**

Both calliper devices and digital photographic methods have been used to quantify foot arch height parameters. The purpose of this study was to compare the reliability of both a calliper device and digital photographic method in determining the arch height index (AHI).

**Methods:**

Twenty subjects underwent measurements of AHI on two separate days. On each day, AHI measurements during both sitting and standing were taken using the AHIMS and digital photographic methods by the same single tester. The intra-tester reliability of each measurement technique was assessed using intraclass correlation coefficients (ICC) and standard error of measurement (SEM). Additionally, the relationship between AHI measurements derived from the two different methods was assessed using a correlation analysis.

**Results:**

The reliability for both the AHIMS and digital photographic methods was excellent with ICC values exceeding 0.86 and SEM values of less than 0.009 for the AHI. Moreover, the reliability of both measurement techniques was equivalent. There was a strong positive correlation between the AHI values collected using both methods. AHI values calculated using the digital photographic method tended to be greater than those derived using the AHIMS.

**Conclusion:**

Digital photographic methods offer equivalent intra-tester reliability to previously established calliper methods when assessing AHI. While AHI measurements calculated using both methods were highly related, the greater AHI values in the photographic method implied caution should be exercised when comparing absolute values between the two methods. Future studies are required to determine whether digital photographic methods can be developed with improved validity.

## Background

The foot is the site at which external forces are applied to the body. Since the foot then transfers these loads further up the kinetic chain, its structure has often been studied in relation to overuse injuries of the lower extremity [1-3]. In particular, the height of the medial longitudinal arch has become a common measurement used to classify foot structure [4-7].

While radiographic measurements are the gold standard in determining the bony structure of the foot, many research laboratories do not have access to such methods. The arch height index (AHI) was developed by Williams and McClay [6] to quantify the height of the arch using handheld callipers. Briefly the AHI is calculated by dividing the height of the dorsum by the truncated foot length (distance from the heel to the first metatarsal head). Although the measurements were stated to be somewhat awkward when performed using handheld callipers, the development of the arch height index measurement system (AHIMS), a mechanical device, improved the ease of taking measurements [8,9]. The measurements of AHI taken using a mechanical device have demonstrated good intra- and inter-tester reliability [8], in addition to validity when compared with equivalent radiographic measurements [6]. However, the reliability has only been quantified using intraclass correlation coefficients. Expressing reliability measurements in terms of coefficients makes it difficult to clinically interpret the results, since the reported reliability units are different from the units of the variable of interest [10]. Therefore, it is desirable to also report reliability within the context of the intended clinical units.

While devices such as the AHIMS have been shown to be reliable and valid, they can be costly to buy or construct. An alternative idea developed recently involved the use of digital photography to assess the height of the arch [5]. Digital photographic techniques potentially offer a highly practical, convenient and cost effective method of assessing arch structure within a clinical or laboratory setting. Such a technique has been shown to demonstrate good to high levels of intra- and inter-tester reliability as well as validity [5]. However, while the study did include the assessment of dorsum height, the reliability of the AHI was not calculated. Therefore, it is difficult to interpret whether the digital photographic method of assessing arch height is as reliable as the equivalent measurement taken with mechanical calliper devices such as those used by Butler and colleagues [8]. Between-day differences in measurements taken using digital photography may arise from errors in manual digitising and camera placement, in addition to the discrepancies that also afflict calliper measurements such as participant positioning. However, reliability measurements for the digital photographic technique have only been calculated based on one photograph of the subject [5]. Therefore, the effect of participant and camera positioning between measurements has not been assessed and requires investigation.

In summary, methods of quantifying the arch height of the foot have been proposed using either manufactured calliper devices or digital photography. However, it remains unclear whether the two techniques demonstrate similar levels of between-day reliability. Therefore, the purpose of this study was to compare the intra-tester reliability of determining arch height when using both a calliper device and digital photographic methods. These reliability data will provide confirmation as to whether photographic techniques can calculate AHI with similar reliability to existing calliper methods.

## Methods

### Subjects

Twenty subjects (6 males and 14 females) volunteered to participate in the study. Subjects were recruited from the University population and the surrounding community. The mean age of subjects was 29.9 ± 5.8 years with a mean weight of 70.4 ± 11.7 kg. The institutional review board approved the study and all subjects provided written informed consent prior to data collection. Subjects were free from lower-extremity injury at the time of testing.

### Experimental protocol

Each subject visited the laboratory on two separate days to have measurements taken on their right foot. Prior to the collection of the foot measurements on the first visit, the weight of the subject was recorded. On each day, measurements were taken using both the AHIMS and digital photographic methods by the same tester. The tester had six months of experience using the AHIMS within a clinical setting.

**Figure 1:**
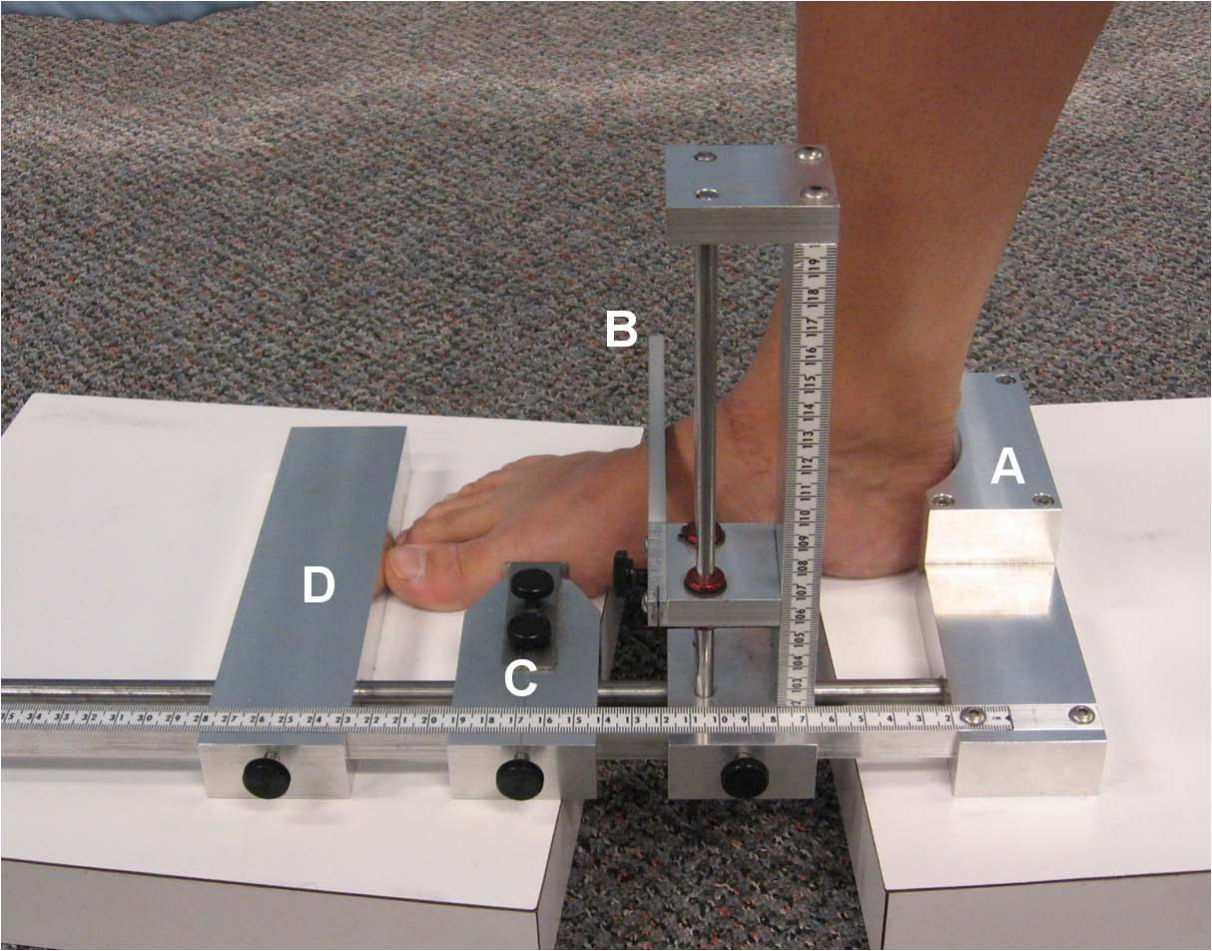
**The Arch height index measurement device (AHIMS)**. The heel is placed against the heel cup (A) and the sliding callipers D and C are aligned against the distal phalanx and first metatarsal head respectively. A third calliper (B) is lowered to the dorsal arch at 50% of the FL.

A portable instrument for measuring the AHI was custom-built based on the AHIMS developed by Richards et al. [9]. This device consisted of a heel cup and series of sliding callipers and rulers (Figure 1). Subjects began seated with their right hip, knee and ankle joints at 90°. Two blocks (thickness = 4.5 cm) were placed under the heel and metatarsal heads of the right foot leaving the arch unsupported. The left foot was placed 15 cm medial to the right foot on a weighing scale (thickness = 4.5 cm) so that the distal end of the hallux of the left foot was positioned 5 cm behind the heel of the right foot. This ensured a clear view of the medial aspect of the right foot which was required for the digital photographic method (see below). The AHIMS was then placed so that the heel cup was against the heel of the right foot and sliding horizontal callipers were used to measure the foot length (FL) and truncated foot length (TFL) (distance from the heel to first metatarsal head). A vertical sliding calliper was then positioned at 50% of the FL, and subsequently used to measure the height of the dorsal arch (DH). The AHI was calculated as the ratio DH:TFL [6]. The subject then stood up with their weight equally distributed on both feet (50% WB) and the measurements were repeated. A final set of measurements were also taken with the subject standing with 90% of their body weight distributed on the right foot (90% WB). A load of 90% BW on the right foot was achieved by asking subjects to lift their left foot off the weighing scale without leaning to either side, until the scale showed that only 10% BW remained on that foot.

**Figure 2:**
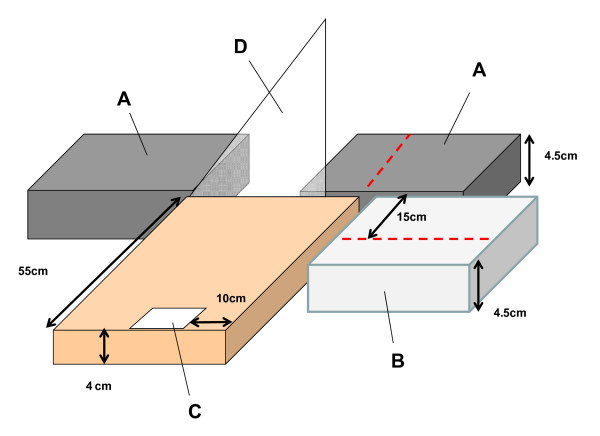
**Setup for the digital photographic method**. The blocks (A) were placed under the heel and ball of the right foot with the medial border lined up with the near edge. The left foot was placed on the scale (B). The camera was placed on another block (C) a fixed distance from the posterior aspect of the heel (10 cm) and medial aspect of the foot (55 cm). A set square (D) was placed in plane with the medial border of the right foot for one of the digital photos to serve as a calibration object.

The digital photographic method involved the same subject set-up as described for the AHIMS. As with the AHIMS, blocks were placed under the right foot with the left foot positioned behind on the weighing scale. A small mark was made on the first metatarsal head to enable the identification of this landmark in the photos. A digital camera (Model Powershot A540, Canon, Tokyo, Japan) was positioned on a block (height = 4 cm) at a fixed distance of 55 cm from the medial border of the right foot and 10 cm forward of the back of the heel (Figure 2). The foot to camera distance was selected based on pilot testing to ensure that the largest expected foot size could be photographed (men's size 13.5 UK). A calibration photo was first taken where an object with known distances (10 cm) was positioned in the plane of the medial arch (55 cm from the camera). The centre of the calibration object was horizontally located approximately perpendicular to the line of view of the camera lens. The calibration object was removed and photos were then taken of the medial aspect of the foot during both sitting (10% WB) and relaxed standing (50% and 90% WB).

**Figure 3:**
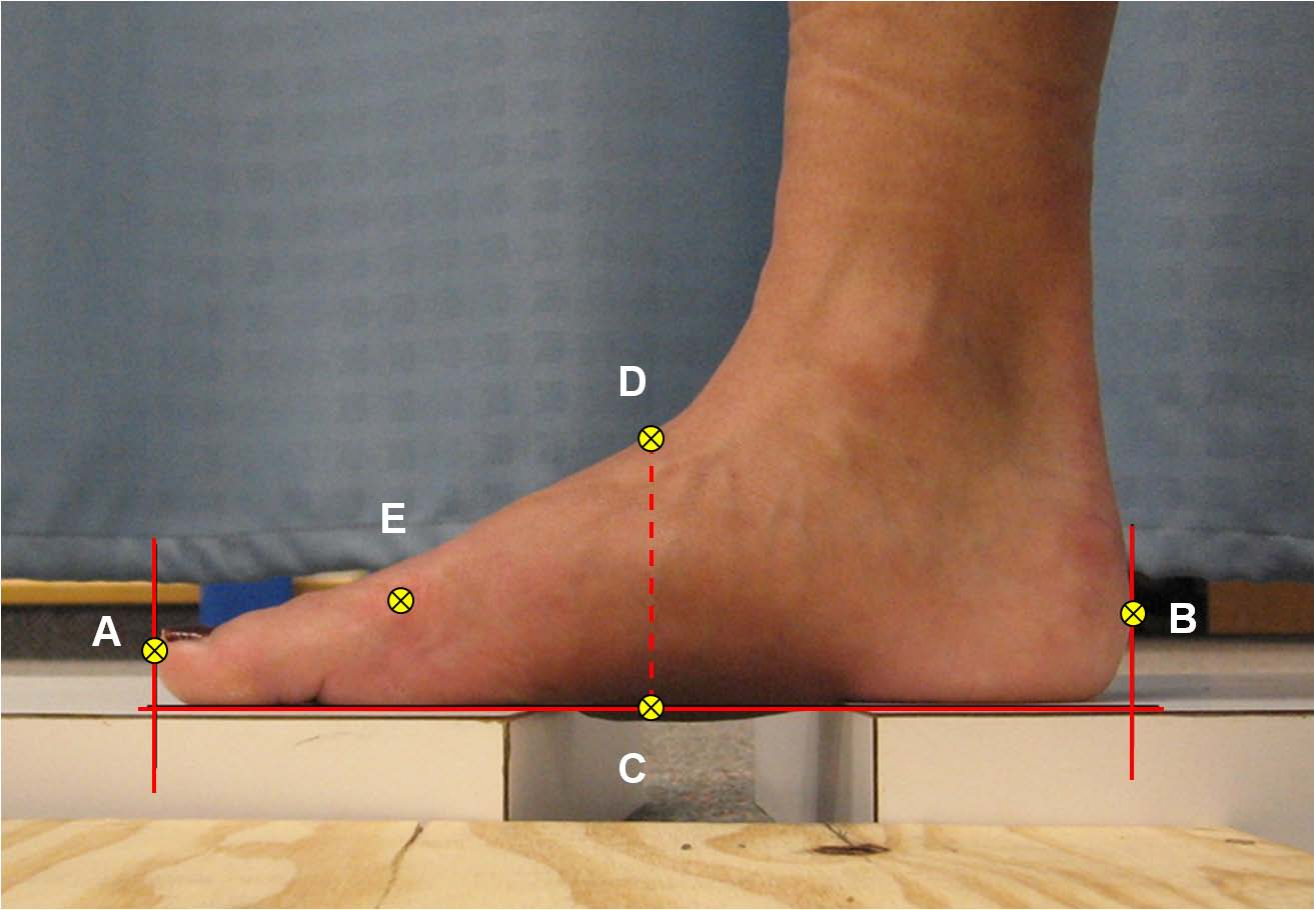
**Digital photographic image used to calculate FL, TFL and AH**. Lines were drawn on the image indicating the distal end of the hallux, the most posterior aspect of the posterior heel, and the horizontal supporting surface. The co-ordinates A-E were digitized and used to calculate the foot measurements. The horizontal distance between A and B gave FL. Point C was placed at the horizontal midpoint between A and B. The vertical distance between C and D represents AH. The horizontal distance between B and E yielded the TFL.

All digital photos were then downloaded onto a PC where they were processed using ImageJ software (NIH, Bethesda, USA). Briefly, this software allowed the digitizing of selected co-ordinates to calculate the foot measurements needed to determine AHI (Figure 3). Co-ordinates were exported from the software as pixels and the calibration photo allowed the conversion of pixels to cm. To assist with the digitizing of the foot photos lines were drawn on the image indicating the distal end of the hallux, the most posterior aspect of the posterior heel, and the horizontal supporting surface (Figure 3). The FL was obtained by digitizing points at the distal end of the hallux and posterior aspect of the heel. The total foot length was then halved to determine 50% of the total foot length. An additional vertical line was then drawn perpendicular from the supporting surface to the dorsum of the foot at 50% of the foot length. The DH was determined by digitizing co-ordinates at the top and bottom of this line. Finally, a co-ordinate on the first metatarsal head was digitized to enable the calculation of TFL. No enhancements or modifications were made to any of the digital images.

### Data analysis

To compare the intra-tester reliability of both the AHIMS and photo methods, intraclass correlation coefficients (ICC 3,1) were calculated for the between-day measurements of both techniques [11]. In addition to ICC values, the between-day standard error of measurement (SEM) was also calculated for each method [12]. Both ICC and SEM were calculated for the variables AHI, TFL and DH. All ICC and SEM reliability variables were assessed during both sitting and standing. Additionally, the relationship between AHI as measured by the AHIMS and photo methods was examined. A Spearman's rank order correlation was performed between AHI (AHIMS) and AHI (digital photo) during standing (50% WB).

## Results

Descriptive statistical values for TFL, DH and AHI for both the AHIMS and photo methods are presented in Table 1. For both measurement techniques, the AHI lowered from sitting to standing. However, there was little difference between the 50% WB and 90% WB standing conditions, with only a 0.004 change in AHI measured. Therefore, reliability data were only presented for the sitting and 50% WB standing conditions.

**Table 1:** Mean and standard deviation (SD) values of truncated foot length (TFL), dorsum height (DH) and arch height index (AHI) for both the AHIMS and digital photographic techniques.

	AHIMS		Digital Photo	
	Mean	SD	Mean	SD
**Sitting (10% WB)**				
TFL (cm)	18.1	0.8	17.4	0.8
DH (cm)	6.8	0.5	6.7	0.5
AHI	0.375	0.020	0.384	0.023
**Standing (50% WB)**				
TFL	18.4	0.8	17.7	0.8
DH	6.3	0.6	6.4	0.6
AHI	0.345	0.025	0.361	0.025
**Standing (90% WB)**				
TFL	18.4	0.8	17.8	0.8
DH	6.3	0.5	6.4	0.6
AHI	0.342	0.024	0.357	0.028

The intra-tester reliability values for foot arch measurements using both methods are shown in Table 2. The mean absolute difference for between-day AHI measurements was less than 0.009 and similar for both the AHIMS and digital photographic techniques. There were no discernible differences between the two measurement techniques in terms of either SEM or ICC values, with both demonstrating excellent reliability. ICCs were in excess of 0.86 and SEM values for the foot measurements used to calculate AHI (TFL and DH) were equal to or less than 0.2 cm.

**Table 2:** Between-day mean absolute differences, standard error of measurement (SEM) and intraclass correlation coefficients (ICC) for both measurement techniques.

	AHIMS			Digital Photo		
	Mean difference	SEM	ICC	Mean difference	SEM	ICC
**Sitting (10% WB)**						
TFL	0.2	0.2	0.94	0.3	0.2	0.91
DH	0.1	0.1	0.94	0.1	0.1	0.93
AHI	0.009	0.009	0.87	0.008	0.008	0.88
**Standing (50% WB)**						
TFL	0.3	0.2	0.93	0.3	0.2	0.92
DH	0.2	0.2	0.94	0.1	0.1	0.95
AHI	0.008	0.007	0.92	0.007	0.006	0.94

The results of the Spearman's rank order correlation suggested there was a strong positive relationship between AHI measurements collected using AHIMS and photographic methods (p < 0.00, ρ = 0.90). The individual subject rankings of AHI (low to high) for each method (AHIMS v digital photo) are listed in Table 3. The absolute difference between the two ranks was ≤ 2 in 16 out of 20 subjects. In general, the AHI values found using the digital photos were greater than the values measured using the AHIMS (Tables 1 and 3).

**Table 3:** Individual subject rankings based on AHI during 50% WB.

Subject	AHIMS		Digital Photo		Rank Difference
	AHI Value	Rank	AHI Value	Rank	
D	0.309	**1**	0.353	**1**	0
J	0.309	**2**	0.363	**4**	-2
P	0.310	**3**	0.380	**5**	-2
O	0.318	**4**	0.320	**2**	2
S	0.322	**5**	0.401	**3**	2
L	0.328	**6**	0.359	**7**	-1
B	0.330	**7**	0.384	**8**	-1
H	0.337	**8**	0.335	**6**	2
A	0.339	**9**	0.379	**11**	-2
C	0.340	**10**	0.333	**15**	-5
F	0.344	**11**	0.397	**10**	1
N	0.347	**12**	0.349	**9**	3
I	0.354	**13**	0.389	**14**	-1
R	0.361	**14**	0.356	**12**	2
E	0.365	**15**	0.323	**20**	-5
T	0.371	**16**	0.335	**16**	0
K	0.372	**17**	0.378	**19**	-2
Q	0.378	**18**	0.372	**13**	5
G	0.379	**19**	0.330	**17**	2
M	0.382	**20**	0.383	**18**	2

Subjects are listed sequentially from lowest to highest values of AHI as measured using the AHIMS. The numerical rank of each subjects' AHI is also listed for the digital photographic method alongside the AHIMS rank. The rank difference was calculated the digital photographic rank subtracted from the AHIMS rank.

## Discussion

The purpose of this study was to compare the intra-tester reliability of two different methods of assessing static arch measurements. The results suggest that arch measurements calculated using a digital photographic method were of a similar reliability to the same variables derived using a mechanical callipers device (AHIMS). Moreover, both methods demonstrated a high level of reliability when calculating AHI with ICC's exceeding 0.86 and SEM's below 0.009.

The ICC values of TFL, DH and AHI measured using the AHIMS were in agreement with previous studies that reported ICC's ranging from 0.91 to 0.99. This provides further confirmation that arch measurements can be collected with excellent reliability when using mechanical calliper devices. The mean AHI value collected during standing using the AHIMS was also similar to the mean values reported in the literature using a similar device [7-9]. However, this value was considerably greater than the mean value of 0.292 reported by Williams and McClay [6]. Butler and colleagues [8] postulated that their mean value of 0.340 was greater than that of Williams and McClay [6] due to the two respective studies collecting standing AHI using different amounts of body weight applied to the measured foot (50% WB versus 90% WB respectively). However, the present investigation found no differences between AHI when measured during 50% WB or 90% WB, thus indicating that the two loading conditions produce a similar measurement outcome.

Although values for AHI have not been reported for the digital photographic method before, the good reliability values for dorsum height are in agreement with McPoil and colleagues [5]. However, given that McPoil et al. [5] did not reposition the participant when assessing reliability, we were curious to explore this further. Indeed, the high reliability of the foot measurements in the present study confirms that the effect of participant positioning between testing sessions was minimal. Moreover, the ICC and SEM values for all foot variables were equivalent to those measured using the AHIMS. This implies that within the context of a single laboratory, a digital photographic method may be used to measure AHI reliably in the absence of mechanical callipers. This is beneficial given that custom built calliper devices can be expensive to construct compared to the cost of a digital camera.

There was a strong correlation between AHI measurements taken using AHIMS and digital photographic methods. Thus, individuals with high and low arches are likely to be identified correctly using either measurement technique. It is perhaps not surprising that both methods were highly correlated since they have both been shown to be highly correlated with equivalent radiographic measurements [5,6]. However, it was noted that mean AHI values measured using digital photos were of a greater magnitude than those recorded using the AHIMS. From the results in Table 1, it would appear that this systematic offset was the result of a shorter TFL being measured in the digital photo method since DH was similar between the two techniques. It is possible that this was the result of the TFL distance (17-20 cm) exceeding the dimensions of the calibration object (10 cm) which might introduce some calibration error. The improvement of calibration procedures such as calibrating over a greater horizontal distance or even using multiple calibration objects, has the potential to increase the validity of TFL measurements conducted using a digital cameras. Given that the digital photographic method was highly correlated with the AHIMS in terms of AHI, it could be speculated that establishing a different set of norms for the photographic method might be a feasible solution. However, a clinical measurement tool such as AHI is much more useful when results can be confidently compared between multiple clinical and research centres. It is presently unknown how equipment and experimental setup might influence the foot variables derived from the digital photos. While good agreement of AHI values between different laboratories has been reported using the AHIMS [9], inter-laboratory comparisons have not been conducted using digital photographic methods. Studies comparing the results from different laboratories and clinics are warranted, in addition to investigating the influence of different camera placements and calibration procedures.

There were some limitations with the current study. Firstly, we only collected intra-tester reliability data. Therefore, it remains to be seen whether the findings can be generalised between different testers. However, strong inter-tester reliability has been reported previously for both the AHIMS [6,8] and digital photographic method [5]. Secondly, it is worth noting that the subjects used in the present investigation were lean and asymptomatic with no notable foot deformities. In cases of pathology, the presence of swelling and deformity may introduce potential error in both the reliability and validity of the measurements taken using both methods. Future work is needed to determine the feasibility of using the AHI measurement in patients with clinical foot pathologies.

## Conclusion

In summary, this study demonstrated that AHI calculated using a digital photographic method can be determined reliably. Moreover, this variable can be obtained with equivalent reliability to a previously established method using mechanical callipers. However, AHI values measured using digital photos were of a greater magnitude than those recorded using callipers. Therefore, future studies are needed to establish whether the digital photographic method can be utilised validly for between laboratory/clinic comparisons.

## Competing interests

The authors declare that they have no competing interests.

## Authors' contributions

MBP developed the rationale for the study. MBP and LF designed the study protocol. LF conducted the data collections and MBP analysed the data. MBP and LF drafted the manuscript. All authors have read and approved the final manuscript.
